# Percutaneous elastic intramedullary nailing of metacarpal fractures: Surgical technique and clinical results study

**DOI:** 10.1186/1749-799X-6-37

**Published:** 2011-07-19

**Authors:** Riazuddin Mohammed, Mohamed Z Farook, Kevin Newman

**Affiliations:** 1 Trauma & Orthopaedics, Hywel Dda NHS Trust, Carmarthen, SA31 2AF, UK; 2 Trauma & Orthopaedics, Frimley Park Hospital NHS Foundation Trust, Frimley, GU16 7UJ, UK; 3 Trauma & Orthopaedics, Ashford & St. Peter's NHS Trust, Chertsey, KT16 0PZ, UK

## Abstract

**Background:**

We reviewed our results and complications of using a pre-bent 1.6 mm Kirschner wire (K-wire) for extra-articular metacarpal fractures. The surgical procedure was indicated for angulation at the fracture site in a true lateral radiograph of at least 30 degrees and/or in the presence of a rotatory deformity.

**Methods:**

A single K-wire is pre-bent in a lazy-S fashion with a sharp bend at approximately 5 millimeters and a longer smooth curve bent in the opposite direction. An initial entry point is made at the base of the metacarpal using a 2.5 mm drill by hand. The K-wire is inserted blunt end first in an antegrade manner and the fracture reduced as the wire is passed across the fracture site. With the wire acting as three-point fixation, early mobilisation is commenced at the metacarpo-phalangeal joint in a Futuro hand splint.

The wire is usually removed with pliers post-operatively at four weeks in the fracture clinic.

**Results:**

We studied internal fixation of 18 little finger and 2 ring finger metacarpal fractures from November 2007 to August 2009. The average age of the cohort was 25 years with 3 women and 17 men. The predominant mechanism was a punch injury with 5 diaphyseal and 15 metacarpal neck fractures. The time to surgical intervention was a mean 13 days (range 4 to 28 days). All fractures proceeded to bony union. The wire was extracted at an average of 4.4 weeks (range three to six weeks). At an average follow up of 8 weeks, one fracture had to be revised for failed fixation and three superficial wound infections needed antibiotic treatment.

**Conclusions:**

With this simple and minimally invasive technique performed as day-case surgery, all patients were able to start mobilisation immediately. The general outcome was good hand function with few complications.

## Introduction

Hand injuries are very common resulting frequently in metacarpal and phalangeal fractures [1]. These commonly involve the active and working population especially in adolescents and young adults [2]. Though a majority of these fractures can be treated non-operatively, surgical intervention is indicated for certain intra-articular fractures, displaced and angulated fractures, rotational deformity, multiple injuries, irreducible and unstable dislocations; and those associated with significant soft tissue injury. Older literature quotes higher degrees of acceptable fracture angulation. This has now been challenged with cadaveric studies showing decreased hand function with metacarpal shortening beyond 5 millimeters and angulation beyond 30 degrees [1,3-5]. They conclude that 30 degrees is the upper limit for acceptable final angulation. However any rotation deformity is poorly tolerated and needs correction [6].

Various fixation techniques in use are percutaneous pinning, cerclage wiring, plating, lag screws, tension band wires and external fixators [2,7-16]. Of these Kirschner wire (K-wire) fixation is a popular choice due to the simplicity of the procedure and the minimal soft tissue interference [17]. We describe a technique and results of using a single pre-bent Kirschner wire (K-wire) for extra-articular metacarpal fractures. The wire acts on a three point intramedullary fixation providing adequate stability and promotes early physiotherapy.

## Surgical technique

After thorough clinical history and physical examination, standard radiographs are performed in the anterioposterior (AP), oblique and true lateral views. Fracture angulation beyond 30° and/or any rotational deformity were the indications for surgical intervention. Patients were counselled about pin site complications and care, and the necessity for removal of the pin after evidence of fracture healing. Only a stable fracture configuration (simple transverse or oblique fractures) or those with minimal communition were fixed using this method. Multi-fragmentary fractures, complex injuries or unreliable patients were not included for this treatment but were instead referred to a specialist hand surgeon for appropriate management.

A single K-wire is pre-bent in a lazy-S fashion with a mild bend at approximately 5 millimeters and a longer smooth curve bent in the opposite direction. Depending on the metacarpal dimensions, either a 1.6 or a 2.0 millimeter (mm) K-wire is used. Under image intensifier, an initial entry point is made at the base of the involved metacarpal using a 2.5 mm drill wire by hand. A T-piece mounted K-wire is then inserted blunt end first in an ante grade manner into the medullary canal after fracture reduction. The advancing end of the wire in the form of a hockey club can be used to aid reduction of the fracture and the wire is then passed across the fracture site. Final position of the reduction is checked on the fluoroscopy and the wire is cut with the tip left out of the skin. In instances where the patients activities so demand, we leave the wire buried in the wound.

A light dressing is applied and the patient is given advice about pin site care. Gentle range of movements exercises are commenced under the supervision of hand therapists. The wound and fixation are reviewed in a week to ten days. Subsequently the wire is removed around four weeks when radiological evidence of fracture healing is visualised. The wire is usually extracted post-operatively at four weeks, mostly in the outpatient clinic; or in the operating theatre if the wire has been buried. The patient is then discharged after one or two subsequent visits to the clinic.

## Materials and methods

All metacarpal fractures that were stabilised operatively from November 2007 to August 2009 using a single pre-bent K wire were retrospectively reviewed. Patients were identified from theatre logs and data was accumulated from the case notes, operative records, physiotherapy notes, clinic letters and radiographs. Patient demographics including age, sex, occupation, handedness and other associated medical problems were collected. The mechanism of injury was noted and the side involved was clinically examined for rotational deformity. Radiographs measured the angulation at the fracture side and the location of the fracture in the metacarpal. Operative data was collected regarding time to surgical intervention, anaesthetic mode, grade of surgeon, tourniquet time and whether the procedure was performed open or percutaneously. Length of stay in the hospital was documented. Post operatively patients were assessed clinically and radiologicallly. Ranges of movements at the metacarpo phalangeal (MCP) joint and inter phalangeal (IP) joint were assessed as well as the presence of any rotational deformity. Fracture union was confirmed on AP, oblique and lateral hand radiographs. Any complications from the procedure including pin site problems and patient tolerance were noted. The procedure and timing of wire removal was also documented.

## Results

In all eighteen little finger and two ring finger metacarpal fractures performed in twenty patients were available for this study. There were three women and seventeen men in the study group and all but four patients were right hand dominant. The average age of the cohort was 25 years and the dominant side was injured in all but one. Eleven of the patients were actively employed, one was unemployed and the rest were students. The predominant mechanism of the injury in ten patients was a punch injury. Other modes were accidental falls and sports related.

The location of the fracture was five diaphyseal and fifteen metacarpal neck fractures. The mean angulation measured was 50.4° (range 35° to 75°) and in addition, five patients also had rotational deformity. The time to surgical intervention from the injury date was a mean 13 days (range 4 to 28 days). The procedures were performed under short general anaesthetic by a Consultant surgeon or a trainee surgeon under supervision. Two procedures needed mini-open osteotomy and reduction as the fracture was a few weeks old. All the wiring procedures were performed percutaneously. The mean tourniquet time was 23 minutes (range 14-35 minutes).

**Figure 1:**
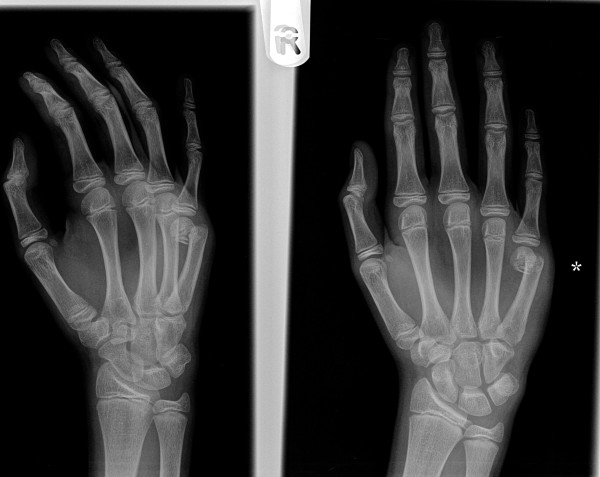
**Radiograph of a 16 year old male patient with displaced little finger metacarpal neck fracture**.

**Figure 2:**
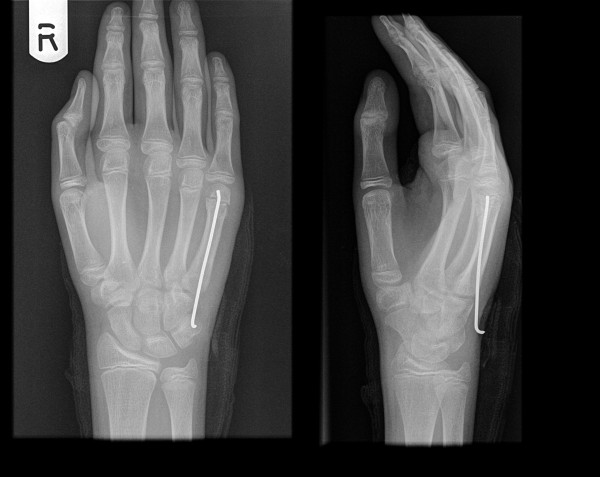
**Metacarpal neck fracture treated with pre-bent K wire**.

**Figure 3:**
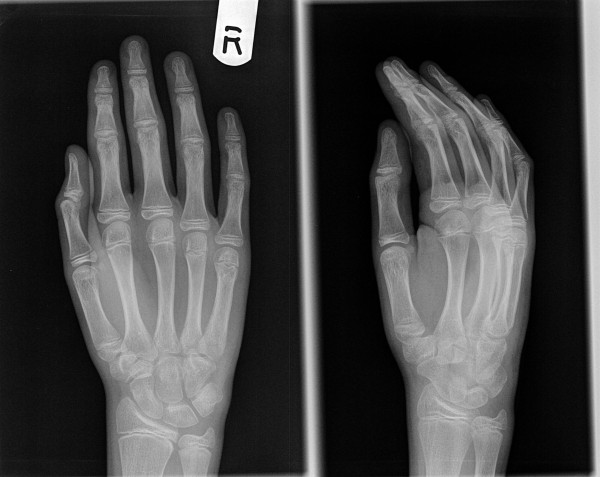
**Radiological evidence of satisfactory outcome after removal of the wire**.

**Figures 4:**
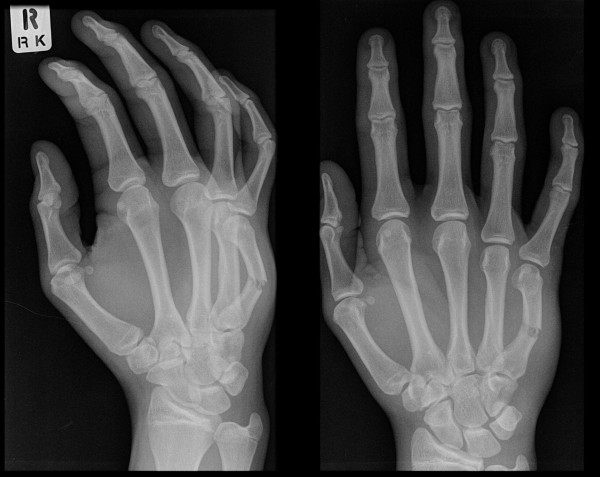
**Pre-operative radiograph of an angulated little finger metacarpal shaft fracture in a skeletally mature adult**.

**Figure 5:**
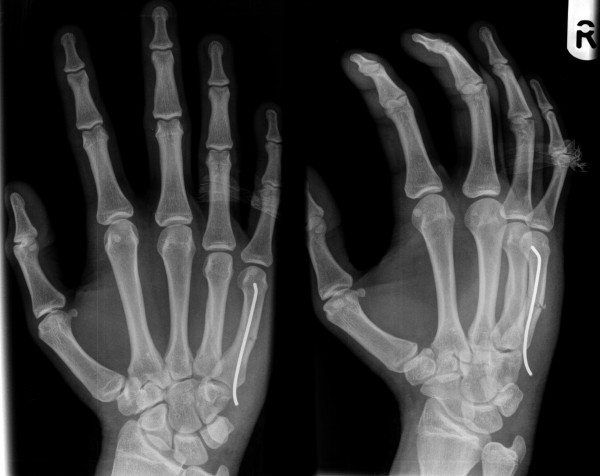
**Post-operative radiograph of satisfactory correction of angulation in the diaphyseal fracture**.

All fractures proceeded to radiological bony union without rotational or severe angulation deformities. (Figures 1, 2, 3, 4 and 5) The wire was extracted in all patients at a mean period of 4.4 weeks (range three to six weeks). Two patients had early (at three weeks) removal of wire for pin site problems. One wire had self buried into the wound and in all five patients had to have the wire removed in the operating theatre. At an average follow up of 8 weeks, one fracture had to be revised for failed fixation and three superficial wound infections with surrounding cellulitis needed antibiotic treatment. All patients regained full flexion at the MCP and IP joints but two patients had a mild extensor lag of about 15°.

## Discussion

Though various methods of internal fixation in metacarpal fractures exist, the principles of treatment include restoration of articular anatomy, stable fixation of fractures, elimination of angular or rotational deformity and rapid restoration of mobility and function. There have been many reports of problems with using plating for these fractures, mainly in relation to the soft tissue impingement [18,19]. The Kirschner wire can be safely used to reduce and stabilise metacarpal fractures. The technique of using multiple K wires for metacarpal fractures was introduced by Foucher [17] ("bouquet" osteosynthesis) and is based on Ender's flexible intramedullary pinning [20]. In the metacarpal, it combines the known benefits of intramedullary implants with minimal iatrogenic soft tissue trauma.

We have modified our technique using a single wire of adequate diameter which is pre-bent to act as an elastic support. With the elastic pre-bent wire acting as a three point fixation, adequate stability is achieved to commence early mobilisation. With minimal soft tissue dissection, avoidance of periosteal stripping and flexible fixation as opposed to rigid fixation; abundant periosteal callus is generated encouraging fracture healing [17]. In addition this procedure is relatively simple, with reduced operating times, minimal radiation exposure and can be performed as day case surgery thereby reducing hospital costs [13]. The disadvantages of the wire technique are lack of absolute stability, wire migration, impalement of soft tissues, pin site problems, infection and the necessity for implant removal [17,21].

In our cohort, the majority of patients were fit young adults with active lifestyles. It is therefore essential to address the functional deficit as well as the cosmetic blemish of the injury. Percutaneous pinning provides a minimal surgical incision with correction of the deformity, adequate fracture stabilisation and early mobility to restore good functional outcome. Because of the healing potential of this younger population, it is ideal to operate early or risk fracture malunion if delayed by a few weeks. In our series, both the open reductions were necessary in patients who were close to four weeks from the injury date. Though the outcome was not significantly different, these patients required longer procedures and an overnight inpatient stay.

We aim to leave the wire protruding from the skin for ease of removal in the outpatient clinic. However if the patients activities demand, the wire is cut flush with the bone and is buried to allow wound closure. Wire migration has been reported to be common with this method and Foucher recommends leaving sufficient length of the wire to allow easy secondary removal.

We had two patients with mild extensor lag and both had buried wires. It is worth mentioning that the metacarpal must be perforated laterally so that the extensor mechanism is not impaled by the wire. We also advise using round tip wires to easily track down the medullary cavity without perforating the cortices. The diameter of the wire chosen depends on the bone and should be strong enough to resist minimal forces during early mobilisation. Foucher's bouquet osteosynthesis method was described using three 0.8 mm wires.

We had to revise one fracture as the wire had backed out loosing the reduction at the fracture site. The fracture was in the metacarpal neck region; and in these cases it is imperative to get 'hockey stick' bend in the K wire at the correct length to be able to adequately hold the smaller distal fragment.

Pin site problems are common as with any K wire technique and the patient should be educated about them. All patients in our study except the one requiring revision were generally satisfied about the surgical experience and with advice about pin site care.

Our study has demerits in that few patient numbers are involved and that it is a retrospective analysis; however we have highlighted the merits of a very simple technique that saves operative time, adequately stabilises the metacarpal fracture, promotes early mobilisation, fewer complication rate and in general obtains a satisfactory outcome in the majority of patients. Larger, prospective studies may be required to validate the technique.

## Competing interests

The authors declare that they have no competing interests.

## Authors' contributions

All the authors have made substantive intellectual contributions to this study. All authors have read and approve the final manuscript for submission.

RM was involved in conception and design, acquisition of data, analysis and interpretation of data and been involved in drafting the manuscript.

MZF was associated with conception of the study, data analysis, interpretation of data, partly involved in drafting the manuscript and has given the final approval of the manuscript.

KN was involved in describing the technique and performing/supervising the procedure, revising the manuscript critically and has given final approval of the version to be published.
